# Cyclic olefin homopolymer-based microfluidics for protein crystallization and *in situ* X-ray diffraction

**DOI:** 10.1107/S0907444909021489

**Published:** 2009-08-06

**Authors:** Soheila Emamzadah, Tom J. Petty, Victor De Almeida, Taisuke Nishimura, Jacques Joly, Jean-Luc Ferrer, Thanos D. Halazonetis

**Affiliations:** a Department of Molecular Biology, University of Geneva, CH-1205 Geneva, Switzerland; b Department of Biochemistry, University of Geneva, CH-1205 Geneva, Switzerland; c Biomedical Graduate Studies Genomics and Computational Biology Group, University of Pennsylvania, Philadelphia, PA 19104, USA; d Department of Plant Biology, University of Geneva, CH-1205 Geneva, Switzerland; e Institut de Biologie Structurale J.-P. Ebel, CEA–CNRS–University J. Fourier, 38027 Grenoble CEDEX 1, France

**Keywords:** cyclic olefin homopolymers, microfluidics, crystallization, *in situ* X-ray diffraction

## Abstract

Microfluidics is a promising technology for the rapid iden­tification of protein crystallization conditions. However, most of the existing systems utilize silicone elastomers as the chip material which, despite its many benefits, is highly permeable to water vapour. This limits the time available for protein crystallization to less than a week. Here, the use of a cyclic olefin homopolymer-based microfluidics system for protein crystallization and *in situ* X-ray diffraction is described. Liquid handling in this system is performed in 2 mm thin transparent cards which contain 500 chambers, each with a volume of 320 nl. Microbatch, vapour-diffusion and free-interface diffusion protocols for protein crystallization were implemented and crystals were obtained of a number of proteins, including chicken lysozyme, bovine trypsin, a human p53 protein containing both the DNA-binding and oligomerization domains bound to DNA and a functionally important domain of *Arabidopsis* Morpheus’ molecule 1 (MOM1). The latter two polypeptides have not been crystallized previously. For X-ray diffraction analysis, either the cards were opened to allow mounting of the crystals on loops or the crystals were exposed to X-rays *in situ*. For lysozyme, an entire X-ray diffraction data set at 1.5 Å resolution was collected without removing the crystal from the card. Thus, cyclic olefin homopolymer-based microfluidics systems have the potential to further automate protein crystallization and structural genomics efforts.

## Abstract 2

A cyclic olefin homopolymer-based microfluidics system has been established for protein crystallization and *in situ* X-ray diffraction.

## 1. Introduction

Crystallization of proteins and determination of their three-dimensional structure provides biological information that is often critical to understanding their function. Indeed, it has been proposed that the three-dimensional structures of all proteins should be solved and the term ‘structural genomics’ has been used for these efforts. So far, these efforts have met with mixed success (Service, 2002 ▶; Chandonia & Brenner, 2006 ▶), in part because protein crystallization is a tedious and time-consuming process that is not easily amenable to automation. Nevertheless, progress towards automation has been made and currently many crystallographers rely on some level of automation for their daily experiments.

Perhaps the most widely used systems for automating pro­tein crystallization are pipetting/robotic systems that simply recapitulate the steps performed by humans (Hui & Edwards, 2003 ▶). One set of pipetting systems prepares crystallization reactions by mixing a precipitant solution with the protein to be crystallized in very small drops (about 200 nl in volume). These drops are placed in chambers containing a much larger volume of precipitant solution. Through vapour diffusion, the volume of the protein drop slowly decreases, leading to pro­tein crystallization. This type of pipetting system has gained acceptance because vapour diffusion is a well established method of protein crystallization (Hui & Edwards, 2003 ▶; Chayen & Saridakis, 2008 ▶) and because it utilizes about ten times less protein than would be required if the same reactions were set up manually. However, a disadvantage of pipetting systems is that the small volume of the protein–precipitant drops leads to significant water evaporation before the chambers are sealed. The degree of evaporation can vary from drop to drop, creating heterogeneity in the experiment, and even occurs when the reactions are prepared in a humidified environment. Robotic systems have also been developed to identify crystals in protein–precipitant drops; these systems consist of a cabinet, in which crystallization plates are stored, a robotic arm and a microscope (Hui & Edwards, 2003 ▶). Several images are acquired from each drop (at various focal planes, since the drops are not flat) and these images are then pro­cessed by software that attempts to identify protein crystals, which is a difficult task because the drop geometry leads to poor images. Finally, a robotic system has also been developed to position crystallization trays in the path of a synchrotron X-­ray beam (Jacquamet *et al.*, 2004 ▶). This system can screen crystals for their ability to diffract X-­rays and allows some crystal parameters, such as space group and unit-cell size, to be determined without manual handling of the crystals.

As an alternative to the systems described above, efforts have been made to use microfluidics for protein crystallization. Several such systems have been developed, most of them employing chips made of elastic silicone (Hansen & Quake, 2003 ▶). In one system, the chips contain wells that are connected by a channel; the elastomeric nature of the chips permits the channel to be sealed by applying mechanical pressure to the chip (Hansen *et al.*, 2002 ▶, 2004 ▶, 2006 ▶). In its most simple form, two wells are filled with protein and precipitant solution, respectively; the pressure on the channel connecting these two wells is then released, allowing the contents of the wells to mix by a process called free-interface diffusion. Depending on the viscocity of the liquids, equilibration of the contents of the two connected chambers is achieved in as little as 1 h. Crystal growth is followed over a period of a few days, but generally for less than a week. The observation time is limited by evaporation of water through the silicone, which is intrinsic to the nature of these microfluidic chips because the silicone elastomer is permeable to water vapour. On the other hand, water evaporation also offers the advantage that it results in higher protein–precipitant concentrations, which may favour crystallization.

Another microfluidics system mixes protein and precipitant in nanolitre-volume droplets which are formed within water-immiscible fluids flowing inside capillary channels (Zheng *et al.*, 2003 ▶, 2004 ▶; Li *et al.*, 2006 ▶). The droplets initially form in silicone elastomer channels, but are eventually guided into glass or Teflon capillary tubes, which are then sealed to prevent evaporation. Depending on the nature of the fluid separating the droplets, this system crystallizes proteins by microbatch or vapour-diffusion methods. When the oil separating the droplets is impermeable to water, the proteins crystallize by the microbatch method. For vapour diffusion, protein–precipitant droplets alternate with droplets containing high concentrations of salt and are separated by a water-permeable oil; this allows the slow transfer of water from the protein–precipitant droplets to the high-salt droplets, resulting in a vapour-diffusion effect.

The use of silicone elastomer is prevalent in microfluidics systems and offers certain advantages, as described above. However, the water-vapour permeability of silicone limits its use in cases where protein crystallization requires incubation with precipitant for more than a few days. Thus, microfluidics systems that utilize vapour-impermeable chips could provide an alternative to silicone elastomer-based systems. Here, we describe our experience with a microfluidics system that uses cyclic olefin homopolymer (COP) as the chip material. We demonstrate the crystallization of several proteins at 277 K and at room temperature using microbatch, vapour-diffusion and free-interface diffusion protocols.

## 2. Experimental procedures

### 2.1. Protein-sample preparation

Chicken egg-white lysozyme (gene accession code NM_205281) and bovine pancreatic trypsin (gene accession code NM_001113727) were purchased as lyophilized powders from Sigma–Aldrich (St Louis, Missouri, USA) and AppliChem (Darmstadt, Germany), respectively. Lysozyme (140 mg ml^−1^) was resuspended in 50 m*M* sodium acetate pH 4.5, whereas trypsin (80 mg ml^−1^) was resuspended in 25 m*M* HEPES pH 7.0, 10 m*M* calcium chloride, 10 mg ml^−1^ benzamidine hydrochloride. A polypeptide consisting of residues 94–291 of human p53 (gene accession code NM_000546) fused to residues 322–356 was expressed in *Escherichia coli*. The cells were lysed in a buffer consisting of 25 m*M* bis-tris propane (BTP) pH 6.8, 250 m*M* NaCl, 5 m*M* DTT and protease inhibitors and the polypeptide was purified by cation exchange (Sepharose SP column; Pharmacia Biotech, Uppsala, Sweden) and gel filtration (Superdex 200 column; Pharmacia Biotech). After purification, the p53 protein was concentrated to 8 mg ml^−1^ in 25 m*M* bis-tris propane pH 6.8, 50 m*M* NaCl, 5 m*M* DTT buffer. A polypeptide corresponding to amino acids 1699–1814 of *Arabidopsis thaliana* Morpheus’ molecule 1 (MOM1; gene accession code NM_179277) was also expressed in *E. coli*. The cells were lysed in buffer consisting of 25 m*M* MES pH 6.0, 200 m*M* NaCl, 5 m*M* DTT and protease inhibitors; the polypeptide was then purified by cation exchange (Sepharose SP column; Pharmacia Biotech) and gel filtration (Superdex 200 column; Pharmacia Biotech) and concentrated to 6 mg ml^−1^ in lysis buffer.

### 2.2. Protein crystallization

Proteins were crystallized either under standard hanging-drop vapour-diffusion conditions in 48-well plates (Hampton Research, Aliso Viejo, California, USA) or in COP cards using a dedicated microfluidics instrument (SpinX Technologies, Meyrin, Switzerland). Lysozyme and trypsin were crystallized at room temperature and the MOM1 fragment was crystallized at 277 K; human p53 was crystallized in the presence of double-stranded DNA containing a high-affinity p53 DNA-binding site at 277 K at a 1:1.1 protein:DNA molar ratio. The double-stranded DNA was prepared by annealing the following two oligonucleotides: 5′-AGAC GGG CATG TCT GGG CATG TCT CA-3′ and 5′-CTTG AGA CATG CCC AGA CATG CCC GT-3′. The precipitant solutions used for crystallization were as follows: 4–30%(*w*/*v*) PME 5000, 1 *M* sodium chloride, 50 m*M* sodium acetate pH 4.5 for lysozyme; 30%(*w*/*v*) PEG 8000, 0.2 *M* ammonium sulfate, 0.1 *M* sodium cacodylate pH 6.5 for trypsin; Index Screen No. 87 [20%(*w*/*v*) PEG 3350, 0.2 *M* sodium malonate pH 7.0], Index Screen No. 89 [15%(*w*/*v*) PEG 3350, 0.1 *M* succinic acid pH 7.0] and Index Screen No. 90 [20%(*w*/*v*) PEG 3350, 0.2 *M* sodium formate pH 7.0] for p53–DNA complexes and 0.2–0.4 *M* magnesium formate, 0.1 *M* Tris pH 8.5 for MOM1. All crystallization buffers and precipitants were purchased from Hampton Research.

### 2.3. Data collection and processing

All data sets were collected on the FIP-BM30A beamline of the ESRF (Grenoble, France; Roth *et al.*, 2002 ▶). For *in situ* data collection, COP cards containing lysozyme crystals were positioned in the path of the X-ray beam using a robotic arm, as described previously (Jacquamet *et al.*, 2004 ▶). Reflection data were indexed, integrated and scaled using the program *XDS* (Kabsch, 1993 ▶). The crystals formed in space group *P*4_3_2_1_2, with unit-cell parameters *a* = 77.1, *b* = 77.1, *c* = 37.2 Å, and contained one molecule in the asymmetric unit. The coordinates of lysozyme (PDB code 1iee) were used as input for refinement, which was performed with the program *CNS* (Brünger *et al.*, 1998 ▶). The electron-density maps and the protein atoms were visualized using the program *O* (Jones *et al.*, 1991 ▶).

## 3. Results

### 3.1. Principle of operation of a cyclic olefin homopolymer-based microfluidics device

**Figure 1:**
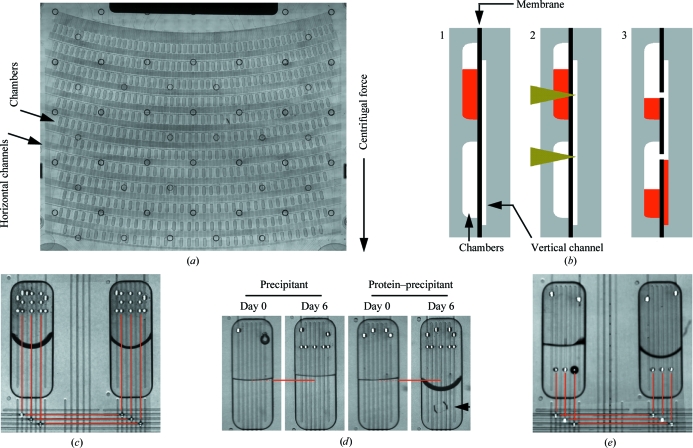
Establishment of protein crystallization protocols in COP microfluidics cards. (*a*) Image of a microfluidics card. Samples are loaded at the top and then move through the card by centrifugal force. (*b*) Diagram of a cross section of a COP card illustrating how defined volumes of liquid are ‘pipetted’. Liquid is contained within a chamber by the thin membrane separating the chambers from the vertical channels (1), holes are opened in the thin membrane by a laser (yellow arrowheads; 2) and the liquid above the hole moves through the vertical channel to a chamber ‘below’ (3). The volume transferred is determined by the vertical position of the hole in the thin membrane. (*c*) Vapour-diffusion protocol. Equal volumes of protein and precipitant were dispensed into one chamber and precipitant only was dispensed into an adjacent chamber. Holes were then opened in the thin membrane above the liquid level to establish connections between the chambers, according to the paths shown by the red lines. (*d*) Changes in liquid volume consistent with vapour diffusion after 6 d of incubation of the COP card at room temperature. The level of liquid at day 0 is indicated by the red lines. The level of liquid in the ‘precipitant’ chamber increases, while the level of liquid in the ‘protein–precipitant’ chamber decreases. In this example, the protein was lysozyme and the black arrow indicates a crystal that formed within 6 d. (*e*) Free-interface diffusion protocol. Protein and precipitant were dispensed into two adjacent chambers. Holes were then opened in the thin membrane below the liquid level to establish connections between the chambers, according to the paths shown by the red lines. All images of individual chambers were acquired using the camera built into the microfluidics instrument. Images showing multiple chambers were assembled from images acquired using an inverted microscope and a low-magnification lens (Zeiss, Gottingen, Germany).

Most microfluidics devices use either silicone elastomers or rigid COPs as the chip material. The vapour-permeability of COPs is several orders of magnitude lower than that of silicone (Mair *et al.*, 2006 ▶), which in theory should make COPs better suited for traditional methods of protein crystallization, where no vapour exchange of the crystallization chamber with the outside environment is desirable (Chayen & Saridakis, 2008 ▶). To explore the potential of COPs in protein crystallization, we used a microfluidics instrument in which the movement and mixing of liquids in COP chips is controlled by centrifugal forces (SpinX Technologies). In this particular system, the microfluidics chip takes the form of a card made of two COP pieces bonded together *via* a thin COP membrane (Figs. 1 ▶ *a* and 1 ▶ *b*). One side of the COP card has chambers arranged in rows and horizontal channels. The chambers have dimensions of 2 × 0.7 × 0.25 mm, corresponding to a volume of about 320 nl. The other side of the COP card contains vertical channels. Connections between chambers and vertical channels, and between vertical and horizontal channels are made by a laser that opens holes in the thin membrane that separates the two sides of the card. Depending on where the openings are made, specified volumes of liquid can be directed from a chamber in one row to a chamber in the row ‘below’ (Fig. 1 ▶ *b*). The movement of the liquids is driven by the centrifugal force generated as the cards are spinning in the instrument.

### 3.2. Establishment of microbatch, vapour-diffusion and free-interface diffusion crystallization protocols

The COP cards used in this study permit the establishment of several protocols for protein crystallization. In the traditional microbatch protocol, protein and precipitant solutions are mixed and the resulting aqueous solution is overlaid with low-density paraffin oil, which is impermeable to water vapour (Chayen & Saridakis, 2008 ▶). This protocol can easily be established in the COP cards simply by directing appropriate volumes of protein and precipitant solutions to the same chamber. Even though the holes that are opened to direct the liquids in the chambers are never sealed, the very small cross-sectional area of the channels results in very small evaporation rates; even after several months the volume of liquid in the chambers does not change appreciably (data not shown).

The second protocol that we established in the COP cards was vapour diffusion (Chayen & Saridakis, 2008 ▶). Protein and precipitant solutions were mixed in one chamber, while an adjacent chamber was filled with precipitant solution only. Connections were then established between these two chambers by opening holes above the liquid level (Fig. 1 ▶ *c*). When the COP cards were incubated at room temperature, changes in the volumes of the liquids in the two chambers consistent with vapour diffusion were observed within 6 d (Fig. 1 ▶ *d*). However, at 277 K vapour diffusion proceeded more slowly, as would be expected.

The third protocol established in the COP cards was free-interface diffusion (Chayen & Saridakis, 2008 ▶). One chamber was filled with protein solution, while an adjacent chamber was filled with precipitant solution. Connections between these two chambers were again established, but in this case by opening holes below the liquid level (Fig. 1 ▶ *e*). For both the vapour-diffusion and free-interface diffusion protocols, the rate of diffusion can be controlled by opening a larger or smaller number of connections between the chambers (between one and five for vapour diffusion and between one and three for free-interface diffusion).

### 3.3. Protein crystallization in COP cards

**Figure 2:**
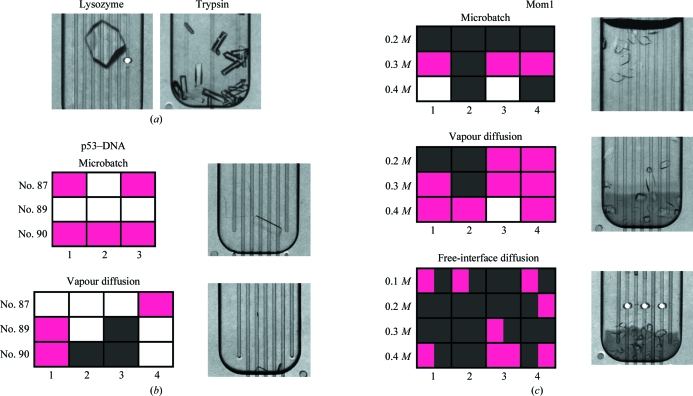
Protein crystallization in COP cards. (*a*) Lysozyme and trypsin crystals formed in COP cards using the microbatch protocol. (*b*) Crystallization of human p53–DNA complexes in COP cards using the microbatch and vapour-diffusion protocols and three precipitant solutions (Index Screen Nos. 87, 89 and 90). Each condition was performed in triplicate or quadriplicate (numbered 1–3 and 1–4, respectively) and the results are colour-coded as follows: protein precipitate, grey; protein crystals, purple; clear solution, white. Examples of the crystals that were formed using each protocol are shown. (*c*) Crystallization of *A. thaliana* MOM1 in COP cards using the microbatch, vapour-diffusion and free-interface diffusion protocols. Each condition was performed in quadriplicate (numbered 1–4) using magnesium formate as the precipitant at the indicated concentrations. The results are colour-coded as described for the p53–DNA complexes in (*b*). For the free-interface diffusion protocol, both the protein (left half) and the precipitant (right half) chambers were scored, since over time both chambers will contain both protein and precipitant. Examples of the crystals formed using each protocol are shown. All images were acquired using the camera built into the microfluidics instrument. The width of the chambers is 750 µm. The detailed compositions of the precipitant solutions are described in §[Sec sec2]2.

The suitability of new protein crystallization platforms is usually documented in the literature using proteins that crystallize readily. Following this tradition, we used the microbatch method to monitor the crystallization of chicken egg-white lysozyme and bovine pancreatic trypsin in the COP cards. For both proteins crystallization was performed in final volumes of 200 nl at room temperature. For lysozyme we implemented a grid of final protein concentrations ranging from 20 to 60 mg ml^−1^ and PME 5000 concentrations ranging from 4 to 30%. Crystals formed at protein concentrations of between 22 and 30 mg ml^−1^ and at PME concentrations of between 18 and 30% (Fig. 2 ▶ *a*). For tryspin, the final protein concentrations ranged from 15 to 40 mg ml^−1^ and PEG 8000 was used at a concentration of 30%. Crystals formed at pro­tein concentrations of between 25 and 30 mg ml^−1^ (Fig. 2 ▶ *a*). Lysozyme and trypsin also crystallized in the COP cards using the vapour-diffusion and free-interface diffusion protocols (Fig. 1 ▶ *d* and data not shown).

Because lysozyme and trypsin crystallize readily, we then studied other proteins that might be more difficult to crystallize. We first focused on the human p53 tumour-suppressor protein. The gene encoding p53 is the most frequently mutated gene in human cancer (Hollstein *et al.*, 1991 ▶). The p53 protein contains a transactivation domain, a sequence-specific DNA-binding domain (residues 94–289) and a homotetramerization domain (residues 325–356). The latter two domains are in­dependently folding domains and their three-dimensional structures have been determined (Cho *et al.*, 1994 ▶; Jeffrey *et al.*, 1995 ▶); however, no structure is available of a p53 polypeptide containing both of these domains. Polypeptides con­taining more than one independently folding domains are generally not easy to crystallize, as the linker between the domains imparts conformational flexibility, which inhibits crystallization. We engineered a p53 polypeptide containing residues 94–291 of human p53 fused to residues 322–356. Based on the boundaries of the crystallized DNA-binding and tetramerization domains, this polypeptide has a flexible interdomain linker that is five amino acids long. Two amino-acid substitutions were introduced in the tetramerization domain of this polypeptide to convert it into a dimerization domain (Davison *et al.*, 2001 ▶). In addition, 13 amino-acid substitutions were introduced in the DNA-binding domain in order to increase its melting temperature and solubility (TJP and TDH, manuscript in preparation). The resulting polypeptide retained its sequence-specific DNA-binding activity. We therefore examined its ability to crystallize in complex with an oligonucleotide containing a p53 DNA-binding site using the microbatch and vapour-diffusion protocols and three different crystallization buffers. Crystals formed with both protocols after 6 d incubation at 277 K in the COP cards. Tabulating the results shows that vapour diffusion yielded p53–DNA crystals with all three crystallization buffers, whereas with the microbatch method p53–DNA crystals were only obtained with two of the three crystallization buffers (Fig. 2 ▶ *b*). The p53 polypeptide–DNA complex also crystallized using the hanging-drop vapour-diffusion method in 48-well plates under the same crystallization conditions (TJP and TDH, manuscript in preparation).

As a second protein that had not been previously crystallized, we focused on *A. thaliana* Morpheus’ molecule 1 (MOM1), a protein that regulates chromatin structure and gene expression without affecting DNA and histone methyl­ation (Amedeo *et al.*, 2000 ▶; Habu *et al.*, 2006 ▶). An evolution­arily and functionally conserved domain of MOM1 maps to a region approximately between amino acids 1734 and 1815 (Caikovski *et al.*, 2008 ▶). We expressed various MOM1 fragments in *E. coli* and found by systematic deletion analysis that a MOM1 polypeptide corresponding to residues 1699–1814 of the full-length protein is soluble. This polypeptide was purified to homogeneity and examined for crystallization at 277 K by the microbatch, vapour-diffusion and free-interface diffusion methods in COP cards, varying the concentration of the precipitant from 0.2 to 0.4 *M*. The best results were achieved using the vapour-diffusion protocol (Fig. 2 ▶ *c*). This fragment of MOM1 also crystallized using the hanging-drop vapour-diffusion method in 48-well plates under the same crystallization conditions (data not shown).

### 3.4. Collection of X-ray diffraction data from crystals in COP cards

The crystals that formed in the COP cards could easily be harvested after opening the cards; these crystals could then be cryopreserved, mounted on cryoloops and frozen, thus allowing complete X-ray diffraction data sets to be collected. When there is a need to examine many crystals, the ability to collect X-ray diffraction data while the crystals are still in the COP card could allow significant savings in time and effort. A robotic arm that is able to position crystallization multi-well plates in front of an X-ray beam has already been described (Jacquamet *et al.*, 2004 ▶). By comparison to multi-well plates, the geometry of the COP cards used in this study appears to be well suited for *in situ* X-ray diffraction analysis.

**Figure 3:**
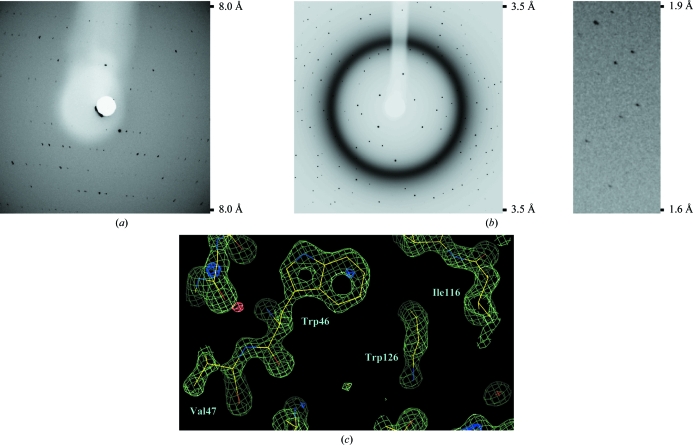
Collection of X-ray diffraction data sets from crystals in COP cards. (*a*) X-ray diffraction pattern of a p53–DNA crystal exposed to the X-ray beam while still in the COP card. The oscillation range was 1°. (*b*) X-ray diffraction patterns of a lysozyme crystal exposed to the X-ray beam while still in the COP card. Two regions of the diffraction image are shown, one encompassing a resolution range lower then 3.5 Å (left) and the other a region from 1.9 to 1.6 Å resolution (right). The oscillation range was 1°. Note that COP absorbs X-rays in the resolution range between 5.4 and 5.1 Å. (*c*) Part of the lysozyme electron-density map contoured at 1.9σ for the 2*F*_o_ − *F*_c_ map (olive green) and at 3σ for the *F*_o_ − *F*_c_ map (dark blue, positive values; orange, negative value). The map shows residues Trp46, Val47 and Ile116 and part of the side chain of Trp126.

To examine whether we could actually collect X-ray diffraction data, COP cards containing p53–DNA, MOM1 and lysozyme crystals were positioned by the robotic arm in the path of the X-ray beam. The robotic arm was programmed to rotate the card during data collection, allowing oscillation of the crystal over a 1° range. For all crystals, we could observe diffraction patterns that were of sufficient quality to allow indexing (Figs. 3 ▶ *a* and 3 ▶ *b* and data not shown). The p53–DNA and MOM1 crystals exposed to X-rays *in situ* diffracted to a somewhat lower resolution level than crystals that had been harvested from the cards, cryopreserved, mounted on loops and frozen. For example, cryopreserved p53–DNA crystals diffracted to 3 Å resolution, whereas the same crystals in COP cards diffracted to about 4.5 Å. We attribute this difference to the temperature shift that occurred during data collection, since the p53–DNA and MOM1 crystals formed at 277 K, whereas the *in situ* data collection was performed at room temperature. In contrast, the lysozyme crystals, which were formed at room temperature, diffracted to a resolution of 1.5 Å when exposed to X-rays through the COP cards (Fig. 3 ▶ *b*).

To evaluate the quality of the data collected from crystals in COP cards, we obtained 45 consecutive X-ray diffraction images, each over an oscillation range of 1°, from a lysozyme crystal. The COP absorbed X-­rays, but only over a narrow resolution range from 5.4 to 5.1 Å (Fig. 3 ▶ *b*). In lower and higher resolution ranges the COP did not compromise data collection, as evidenced both by observing the X-ray diffraction images (Fig. 3 ▶ *b*) and also from the statistics describing the integration of the X-ray reflection intensities over the 45 frames of collected data (Table 1 ▶). Data in the resolution range 40–1.5 Å were used for refinement using a previously determined lysozyme structure as input (Sauter *et al.*, 2001 ▶). The refined structure had excellent statistics (Table 1 ▶) and well resolved electron-density maps (Fig. 3 ▶ *c*), especially considering that data from only 45 frames were used for refinement.

## 4. Discussion

The need to optimize the efficiency with which X-ray diffraction-quality protein crystals are produced has led to the development of methods for automating the setup of protein crystallization reactions and for reducing the amount of pro­tein required (Chayen & Saridakis, 2008 ▶). Most microfluidics systems utilize silicone elastomers as the chip material and have achieved exceptional economies in the amount of protein consumed: in one system, 10 nl protein solution is required per crystallization condition. However, silicone elastomers are also highly permeable to water vapour and this limits their utility to proteins that crystallize within a few days (Hansen & Quake, 2003 ▶). Materials that are impermeable to water vapour have also been explored in protein crystallography at a miniaturized scale, but in general these systems require significant human intervention or are compatible with only one method of protein crystallization, usually free-interface diffusion (Ng *et al.*, 2003 ▶, 2008 ▶). This is because materials that are impermeable to water vapour, such as COPs, are rigid. Unlike chips made of silicone elastomers, in which liquids can be moved by deforming the chip itself, the movement of nanolitre volumes of liquid in rigid chips is not a trivial task. The system we used here solves the ‘pipetting’ problem by opening holes at defined positions to control the volume of liquid to be dispensed and by spinning the cards to move the liquids by centrifugal force. Once the problem of pipetting had been addressed, COP-based microfluidic chips could easily be adapted for protein crystallization using several well established protocols, as demonstrated here.

COP cards may overcome some of the limitations inherent in microfluidics chips made of silicon elastomers. The first is the issue of water-vapour permeability. In COP cards there is very little water evaporation even after months of incubation at room temperature. A second limitation of silicone elastomer chips is that crystals cannot readily be isolated for X-ray diffraction analysis. This means that new protein crystals have to be obtained using traditional protein crystallization methods. In some cases, it is not straightforward to translate the conditions under which proteins crystallize by free-interface diffusion in the microfluidics chip to conditions under which they will crystallize by the traditional hanging-drop vapour-diffusion method in multi-well plates. COP cards overcome this limitation, because the volume of the chambers (320 nl) allows even relatively large protein crystals to form; these crystals can then be easily harvested from the COP cards for the collection of X-ray diffraction data sets. Alternatively, limited diffraction data can also be collected from the crystals *in situ*, because COPs absorb X-rays only within a defined resolution range of about 5.4–5.1 Å (Fig. 3 ▶ *a*; Ng *et al.*, 2008 ▶). In exceptional cases, as illustrated here with the example of lysozyme, entire X-ray diffraction data sets can be collected. However, this was possible with lysozyme because sufficient data could be collected from just 45 images and because the lysozyme crystals did not suffer extensive radiation damage, even though the COP card was at room temperature during data collection. In the case of the MOM1 and p53–DNA crystals only about ten images could be collected per crystal.

COP-based microfluidics systems compare favourably with automated pipetting systems that set up crystallization reactions in multi-well plates (Chayen & Saridakis, 2008 ▶). In the latter systems all pipetting steps are performed in an open environment, which allows water to evaporate while the drops are being set up, especially when the volume of these drops is in the nanolitre range. In contrast, in microfluidics systems all pipetting steps are performed in a closed environment, thus eliminating the problem of water evaporation during setup. Further, the COP cards described here can be stacked in holders, so that their inlets adopt the same geometry as the wells of 384-well plates. Thus, the initial loading of protein samples and precipitant solutions in the COP cards can be performed with standard pipetting robots. A final advantage of the COP cards is the geometry of their chambers, which allows easy visualization of their contents. Thus, protein crystals can be easily identified, a task that is much harder to accomplish with crystals formed in round hanging or sitting drops. Based on our experience, we anticipate that COP-based microfluidics will play an important role in protein crystallization efforts in the near future.

## Figures and Tables

**Table 1:** Data-collection and refinement statistics for a lysozyme data set comprised of 45 consecutive frames, each having an oscillation range of 1° Values in parentheses are for the highest resolution shell.

Data collection	
X-ray wavelength (Å)	0.97958
Space group	*P*4_3_2_1_2
Unit-cell parameters (Å)	*a* = 77.1, *b* = 77.1, *c* = 37.2
Resolution (Å)	40–1.5 (1.59–1.5)
Observations	73037 (11316)
Unique reflections	18458 (2903)
Data coverage (%)	91.7 (91.5)
〈*I*/σ(*I*)〉	12.5 (4.1)
*R*_merge_† (%)	6.8 (31.6)
Refinement statistics	
Resolution range (Å)	40–1.5
Reflections used [>0σ(*F*)]	17529
Protein atoms	1001
Water molecules	110
*R* factor‡ (%)	21.6
*R*_free_§ (%)	23.8
R.m.s. deviations¶	
Bonds (Å)	0.006
Angles (°)	1.264
Ramachandran plot	
Most favoured (%)	85.8
Allowed (%)	14.2
Disallowed (%)	0.0

† *R*_merge_ = 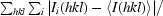 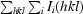 for the intensity (*I*) of *i* observations of reflection *hkl*.

‡ *R* factor = 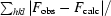 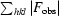, where *F*_obs_ and *F*_calc_ are the observed and calculated structure factors, respectively.

§ *R*_free_ is the *R* factor calculated using 5% of the reflection data chosen randomly and omitted from the start of refinement.

¶ R.m.s. deviations for bonds and angles are the respective root-mean-square deviations from ideal values.
